# Biomechanical effects of different mandibular movements and torque compensations during mandibular advancement with clear aligners: a finite element analysis

**DOI:** 10.3389/fbioe.2024.1496517

**Published:** 2024-11-13

**Authors:** Ya Wang, Baraa Daraqel, Ying Wang, Dan Yang, Yihan Dong, Yun Hu, Leilei Zheng

**Affiliations:** ^1^ Stomatological Hospital of Chongqing Medical University, Chongqing, China; ^2^ Chongqing Key Laboratory of Oral Disease and Biomedical Sciences, Chongqing, China; ^3^ Chongqing Municipal Key Laboratory of Oral Biomedical Engineering of Higher Education, Chongqing, China; ^4^ Department of Orthodontics, Oral Health Research and Promotion Unit, Al-Quds University, Jerusalem, Palestine

**Keywords:** finite element analysis, clear aligner, mandibular advancement, torque compensation, class II malocclusion

## Abstract

**Introduction:**

This study aimed to evaluate the biomechanical effects of different mandibular movements and torque compensations during mandibular advancement with clear aligners using finite element analysis.

**Methods:**

Models were constructed to include the mandible, teeth, periodontal ligament (PDL), and clear aligners with buccal wings. Five oral muscles (superficial masseter, deep masseter, medial temporalis, posterior temporalis, and medial pterygoid) were represented as springs. Muscle values were measured and applied during different mandibular movements, including advancement distances (1–7 mm) and occlusal opening distances (2–4 mm). Different torque compensation angles (0°, 1°, 2°, and 3°) were applied to the mandibular central incisor.

**Results:**

When the mandibular advancement was equal to or slightly excessed the occlusal opening distance, stress on the posterior PDL decreased and became more evenly distributed. Increasing the occlusal opening distance significantly raised stress on the posterior PDL and caused grater labial inclination of the mandibular anterior teeth. As the torque compensation increased, the labial inclination of the mandibular central incisor decreased, but stress on the PDL increased. Nearly complete bodily movement of the lower central incisor was achieved with torque compensation angles of approximately 15°, 19°, and 20° in models M1-2, M2-3, and M3-4, respectively.

**Conclusion:**

To maintain periodontal health during mandibular advancement, it is recommended that the mandibular advancement distance be equal to or slightly excessed the occlusal opening distance. Excessive occlusal opening distance increases stress on the posterior PDL and the labial inclination of mandibular anterior teeth, requiring careful control. Additionally, proper torque control of the mandibular interior teeth is crucial for optimal outcomes.

## 1 Introduction

Class II malocclusion is one of the most prevalent orthodontic conditions, affecting approximately 20% of the global population with permanent dentition (Alhammadi et al., 2018). Skeletal Class II malocclusion is caused by mandibular retrognathia, maxillary protrusion, or a combination of both, with mandibular retrognathia being the most common (McNamara, 1981). In individuals with normal jaw anatomy, mandibular movements, such as opening, closing, and lateral shifts, are typically smooth and well-coordinated, allowing for balanced occlusal contact and function. However, patients with maxillofacial deformities, such as skeletal Class II malocclusion due to mandibular retrognathia, often exhibit altered jaw mechanics. These differences may include restricted mandibular advancement, altered occlusal contact patterns, and imbalanced muscle function, affecting both function and aesthetics (Alshammari et al., 2022). Understanding these variations is crucial when designing orthodontic interventions aimed at correcting such skeletal discrepancies.

Various appliances, including fixed and removable functional appliances like Twin Block, Bionator, Forsus, and Herbst, are frequently used to correct skeletal Class II malocclusions. These appliances have been shown to effectively stimulate mandibular growth, modify the position of the maxillomandibular complex, and improve the soft tissue profile, ultimately contributing to better patient psychosocial well-being (Cardoso et al., 2020; Sharma et al., 2021; Flores-Mir et al., 2018). However, they may also result in unwanted dental side effects, such as labial tipping of mandibular incisors and retrusion of maxillary incisors, which can limit their overall skeletal effects (Bishara, 2006). Recent advancements, such as skeletal anchorage Class II elastics and the use of Forsus with miniscrew or miniplate anchorage, have been developed to reduce the adverse dental side effects associated with conventional appliances (Ozbilek et al., 2017; Celikoglu et al., 2016). In 2017, Align Technology (San Jose, Calif, CA, United States) introduced clear aligner treatment (CAT) with Invisalign^®^ Mandibular Advancement specifically targeting growing patients with skeletal Class II malocclusion due to mandibular retrusion (Meade and Weir, 2024). The effectiveness of CAT for mandibular advancement is well-documented, and it has gained popularity among both clinicians and patients (Ravera et al., 2021; Liu C. et al., 2022; Sun and Liu, 2022). Unlike conventional appliances that rely on a single-stage approach, CAT employs a multi-stage strategy for mandibular advancement (Wang and Liu, 2021).

Clinical studies have highlighted differences in the biomechanical effects of mandibular advancement between traditional functional appliances and CAT (Ravera et al., 2021; Lund and Sandler, 1998; Zybutz et al., 2021). While traditional functional appliances are more effective in reconstructing the mandibular ascending ramus and condyle (Yu et al., 2023), CAT offers better control over the labial inclination of the mandibular anterior teeth and minimizes the clockwise rotation of the mandibular plane compared to conventional methods (Wu et al., 2023; Sabouni et al., 2022; Yang et al., 2021). Despite these benefits, CAT still results in some labial inclination of the lower anterior teeth during mandibular advancement, which may increase the risk of periodontal damage, including gingival recession and bone dehiscence (Morris et al., 2017; Sulewska et al., 2021). Additionally, the impact of CAT on the clockwise rotation of the mandibular plane remains a subject of debate (Yang et al., 2021; Koukou et al., 2022). Currently, there are no clear guidelines regarding the optimal levels of mandibular advancement and occlusal opening when using CAT, and external root resorption continues to be a common side effect of this treatment (Fang et al., 2019; Brezniak and Wasserstein, 1993). Given these challenges, further investigation is needed to determine the appropriate amounts of mandibular advancement and occlusal opening to minimize the risk of periodontal damage during CAT.

In orthodontic treatment, stress distribution is critical because it directly influences tooth movement, periodontal health, and the stability of treatment outcomes. Finite Element Analysis (FEA) is a non-invasive, radiation-free numerical method that enables three-dimensional analysis of tissue physiological responses and stress distributions (Duanmu et al., 2021). It has been applied in orthodontic treatments, including clear aligners, mandibular advancement devices, and Herbst appliances (Yang et al., 2023; Caragiuli et al., 2021; Zhu et al., 2022). As both experimental and clinical studies are needed to better understand the biomechanics of mandibular advancement with clear aligners and to establish more definitive treatment protocols. Therefore, this study employs FEA, combined with simulations of oral muscles, to investigate the biomechanical effects of clear aligners for mandibular advancement under various conditions, as well as to quantitatively analyze torque control on the anterior teeth. These efforts aim to provide new insights and treatment strategies for clinical practice.

## 2 Materials and methods

### 2.1 Construction of an orthodontic model

**FIGURE 1:**
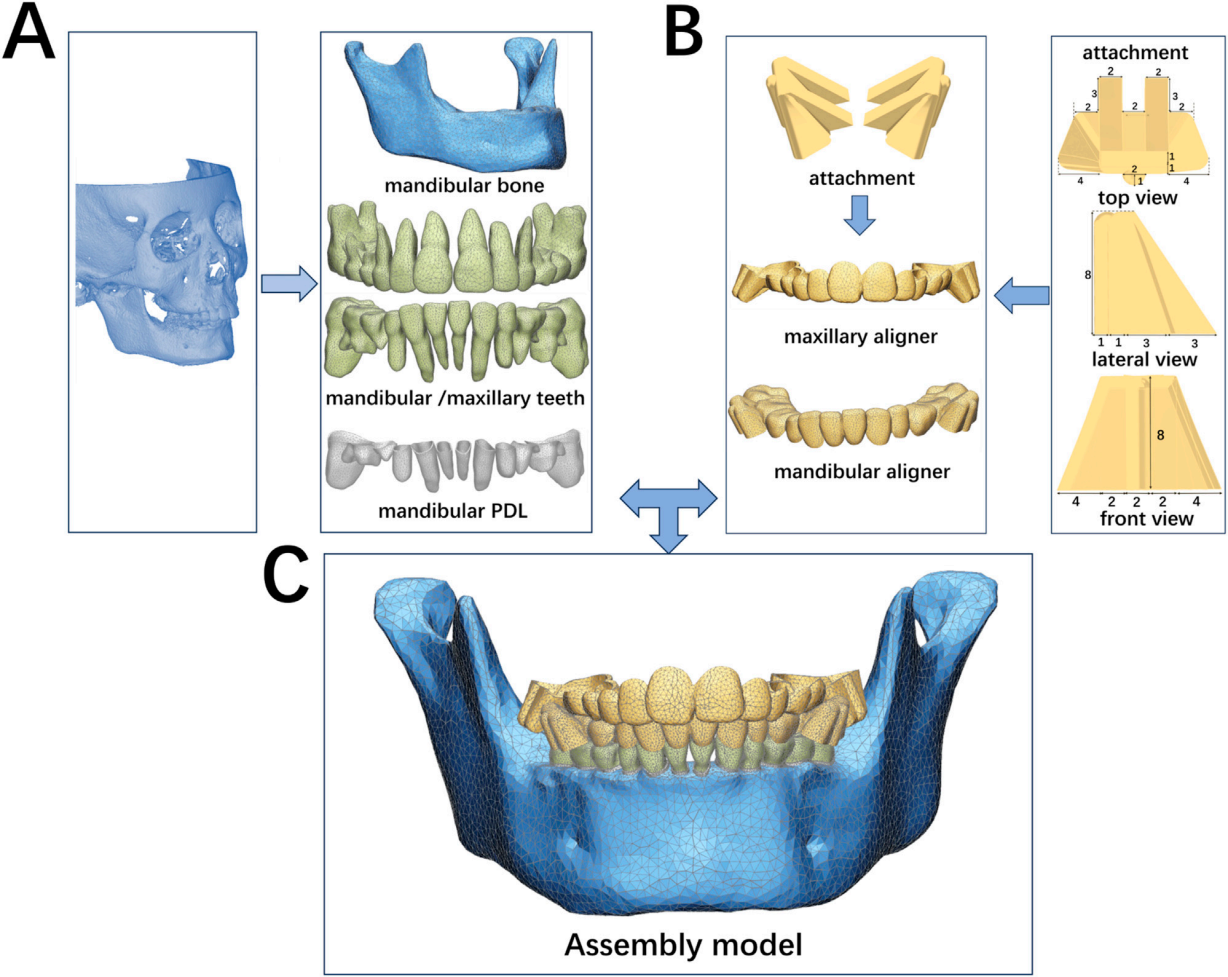
Finite element models. **(A)** Generation of teeth, PDL and the mandible. **(B)** Design and generation of clear aligners with buccal wings; **(C)** Assembly model with unstructured 4-noded tetrahedral elements. This model includes permanent teeth: 31, 32, 36, 41, 42, 46; primary teeth: 73, 74, 75; 83, 84, 85.

This study was approved by the Ethics Committee at the Stomatological Hospital of Chongqing Medical University (2024–104). The patient selected for this study was a healthy 10-year orthodontic patient in the peak growth phase (CVM 3) and mixed dentition stage, presenting with a skeletal Class II malocclusion due to mandibular retrognathia (SNA: 78.1°, SNB:71.1° and ANB:7°). Three-dimensional (3D) models of the maxillary and mandibular bones, as well as teeth, were reconstructed using data from cone-beam computed tomography (CBCT) scans through Mimics Research software (19.0; Materialise, Leuven, Belgium) and Geomagic Warp software (3D Systems, Rock Hill, SC, United States) (Barone et al., 2017). The PDL was modeled by displacing the root surface outward by an average of 0.2 mm (Seo et al., 2021). The mandibular alveolar fossa models were generated by subtracting the mandibular teeth and PDL from the mandible using Boolean operations (Figure 1A) (Liu L. et al., 2022).

Buccal wing attachments were designed to replicate clinical mandibular advancement (Figure 1B). The interdental spaces between the maxillary and mandibular dental arches were labeled as “U” for the upper arch and “L” for the lower arch. These attachments were positioned on the first molar region of the upper arch (“U”) and the second primary molar region of lower arch (“L”). A sagittal space of 1.5 mm and an occlusal space of 1.5 mm were intentionally left between the bilateral buccal wing attachments. The attachments positions were established using SolidWorks software (SolidWorks, United States). The outer surfaces of the tooth crowns and buccal wings were adjusted to create the appliance’s inner surface, aligning with the boundary curves of clear aligner. Clear aligners with buccal wings were created by applying an external offset with a thickness of 0.75 mm (Meng et al., 2019) (Figure 1B).

The mandibular bone, teeth, PDL, and clear aligners with buccal attachments were assembled using 3-Matic software (11.0; Materialise, Leuven, Belgium) to generate a 3D FEA solid model with unstructured 4-noded tetrahedral elements (Figure 1C). Different mesh divisions were applied as follows: 0.5 mm for teeth, 1.5 mm for the mandibular bone, 0.1 mm for the PDL, 0.5 mm for the mandibular aligner, and 1.25 mm for the maxillary aligner. All models were configured to exhibit linear elastic properties (Kojima and Fukui, 2012). The number of nodes and elements is listed in Table 1.

**TABLE 1:** Material properties and number of nodes and elements of the components

Component	Nodes	Elements	Young’s modulus (MPa)	Poisson’s ratio
Bone	172180	98491	13700	0.3
PDL	1603315	830393	0.68	0.49
Teeth	227307	74081	18600	0.31
Clear aligner	244907	128060	816.31	0.3

These models were then imported into ANSYS software (ANSYS Inc., United States) for further analysis. As shown in Table 1, the material properties employed in this study were based on data from the study by Gomez et al., (2015). The mandibular PDLs were tightly bonded to the roots of the mandibular teeth and the supporting alveolar bone. To simulate the mandibular advancement, a “no separation” contact was established between the bilateral buccal wing attachments, allowing for relative sliding without separation (Zhiguo et al., 2023). A friction coefficient of µ = 0.2 was applied between the clear aligners and the corresponding teeth (Yao et al., 2023). The internal surface of maxillary clear aligners was set as a fixed part when the muscles forces were applied.

### 2.2 Design of aligner model

#### 2.2.1 Orders of mandibular movement

A meta-analysis study has demonstrated that the mandibular plane angle remains largely unchanged during mandibular advancement with clear aligners (Yu et al., 2023). In this study, the total sagittal movement of the mandible is referred to as the advancement distance, while the total vertical movement is termed the occlusal opening distance. To investigate the biomechanical effects of different mandibular displacements, clear aligner models were created with occlusal opening distances of 2 mm (M1), 3 mm (M2), and 4 mm (M3). Mandibular advancement was set within a range of 1–7 mm. For example, when the mandibular advancement was 1 mm, the models were designated as M1-1, M2-1, and M3-1, respectively.

#### 2.2.2 Measurement and loading of oral muscles

**FIGURE 2:**
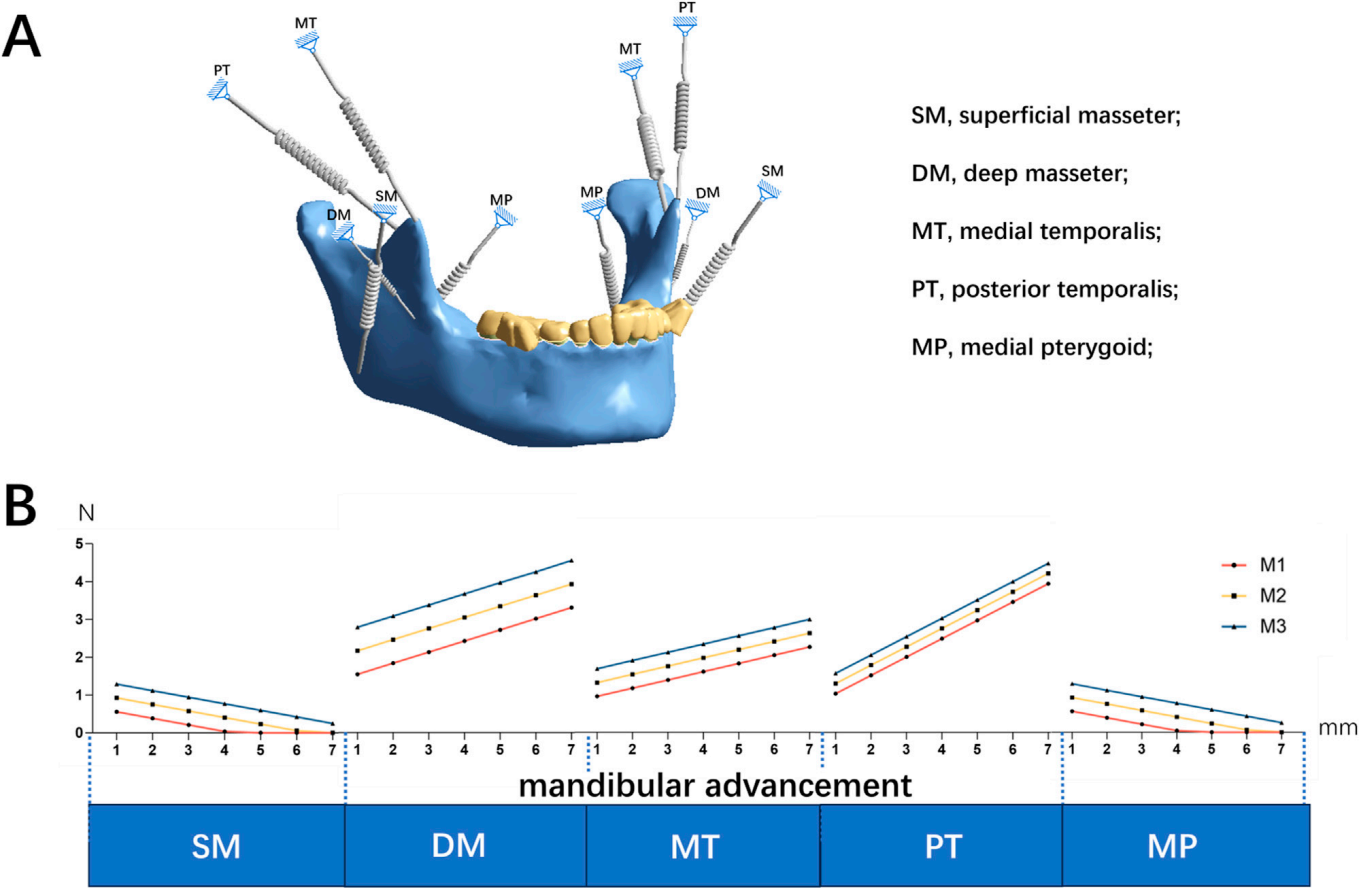
**(A)** The mandibular masticatory muscle activity using spring elements of the numerical model **(B)** Muscle forces in different mandibular occlusal opening distances (2–4 mm, M1 = 2 mm, M2 = 3 mm, M3 = 4 mm) and advancement distances (1–7 mm).

To accurately simulate mandibular advancement while using orthodontic appliance, this study modeled mandibular masticatory muscle activity using spring elements (Guo et al., 2013; Zhou et al., 1999; Pachnicz and Stróżyk, 2021; Ding et al., 2022). During the mandibular advancement, the inferior and superior heads of the lateral pterygoid muscle were kept passive (Zhu et al., 2022). The middle and posterior parts of the temporalis muscle were primarily responsible for restoring force. Therefore, the study included the superficial and deep parts of the masseter (SM, DM), middle and posterior parts of the temporalis (MT, PT), and the medial pterygoid (MP) (Figure 2A). Muscle forces were measured at mandibular occlusal opening distances of 2–4 mm and advancement distances of 1–7 mm. These forces were then applied to the model at muscle attachment sites based on the 3D distribution of muscles following different mandibular movements.

#### 2.2.3 Anterior torque compensation of aligner

To evaluate torque changes during mandibular advancement, various torque compensations (θ = 0°, 1°, 2°, 3°) were applied to the right mandibular central incisor. Given that there were 21 models, with 7 models corresponding to each occlusal opening distance, torque compensations were only applied to those models that resulted in lower and more evenly distributed stress, based on the study’s findings. These specific models were not predetermined in the pre-research planning but were instead chosen during the study as the results indicated their suitability. The target teeth were rotated counterclockwise around their volume center, with the crown facing the lingual side and the root facing the buccal side. Subsequently, new aligners were then created to accommodate the repositioned dentition.

#### 2.2.4 Coordinate system setting

The coordinate system was established using the CBCT framework (Ding et al., 2022). The *x*-axis was defined as the intersection line between the coronal and the occlusal planes, extending from the patient’s right to left side. The *y*-axis was defined as the intersection line between the sagittal and occlusal planes, extending positively from the anterior to the posterior teeth. The *z*-axis was set as the intersection line between the coronal and sagittal planes, extending from bottom to top. Von Mises equivalent stress was used as the standard to measure the stress levels in PDLs and clear aligners. Additionally, the displacement trends of the mandible and mandibular teeth were analyzed.

## 3 Result

The restorative forces during mandibular advancement are illustrated in Figure 2B. As mandibular advancement increased from 1 to 7 mm, the forces generated by SM and MP decreased. In contrast, the forces from the MT, DM, and PT increased, becoming dominant with greater mandibular advancement (Figure 2B). Detailed results regarding the forces and directions of muscle activity at varying advancement distances and occlusal opening distances are provided in the Supplementary File S1.

**FIGURE 3:**
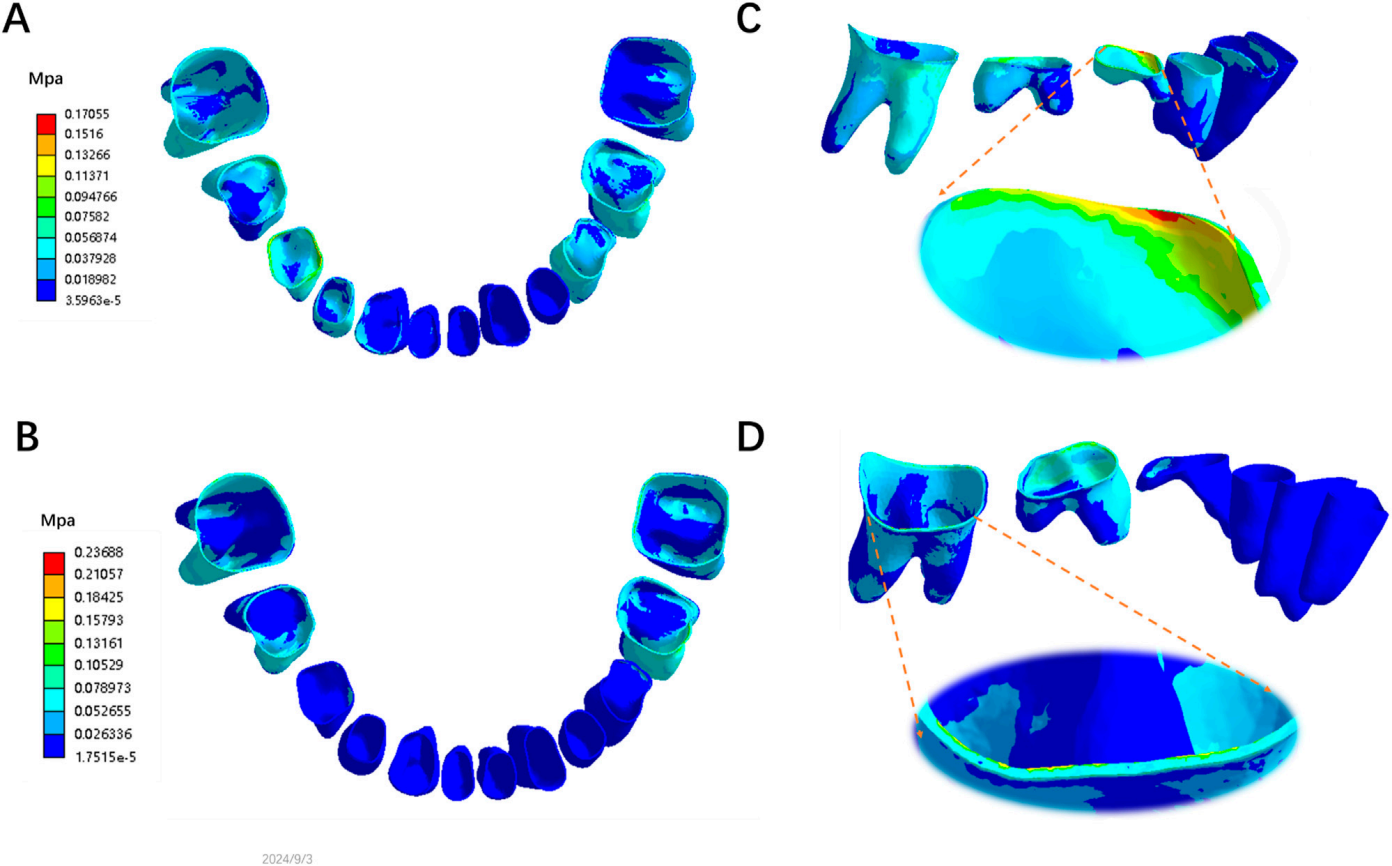
The Von-mises (equivalent) stress distribution on the mandibular PDL on the model M2-1 **(A)** and M2-7 **(B)**; The ratio of advancement distance to occlusal opening distance <1 **(C)** and >1 **(D)**.

**FIGURE 4:**
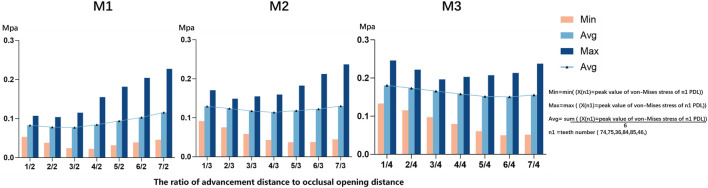
The Von-mises (equivalent) stress of mandibular posterior PDL in different ratio of advancement distance (1–7 mm) to occlusal opening distance (2–4 mm, M1 = 2 mm, M2 = 3 mm, M3 = 4 mm).

Equivalent stress on the PDL was primarily concentrated in the posterior dental region, with relatively lower stress in the anterior dental region (Figures 3A, B). In the posterior dental region, when the ratio of advancement distance to occlusal opening distance was less than 1, stress concentrated in the mandibular first primary molar (Figure 3C). However, as the ratio exceeded 1, the stress shifted to the mandibular first molar (Figure 3D). When the ratio was less than 1, the PDL stress decreased and became more evenly distributed as the ratio increased. Conversely, when the ratio exceeded 1, the PDL stress increased and became more concentrated. Notably, PDL stress was lower and more evenly distributed when the mandibular advancement distance was equal to or slightly greater than the occlusal opening distance (Figure 4).

**FIGURE 5:**
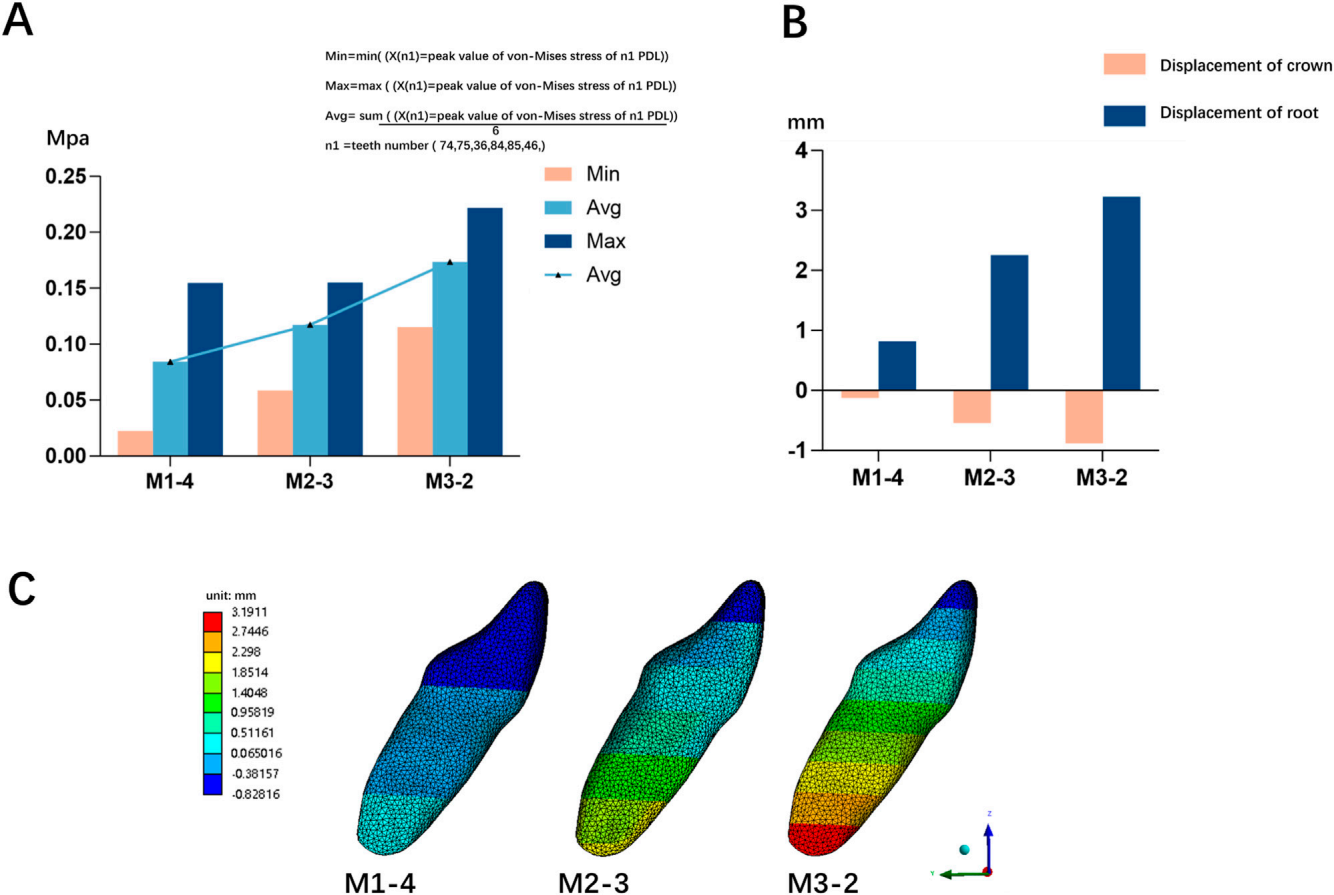
**(A)** The Von-mises (equivalent) stress of mandibular posterior PDL in different occlusal opening groups **(B)** The crown-root movement of mandibular right central incisor in different occlusal opening groups **(C)** The displacement of mandibular right central incisor in different occlusal opening groups; 2/4 (M1), mandibular advancement distance (2 mm) and occlusal opening distance (4 mm); 3/3 (M2), mandibular advancement distance (3 mm) and occlusal opening distance (3 mm); 4/2 (M3), mandibular advancement distance (4 mm) and occlusal opening distance (2 mm).

When the sum of advancement distance and occlusal opening distance was held constant, increasing occlusal opening significantly elevated stress on the posterior PDL and caused labial inclination of mandibular anterior teeth (Figure 5). Conversely, increasing the advancement distance produced the opposite effect.

**FIGURE 6:**
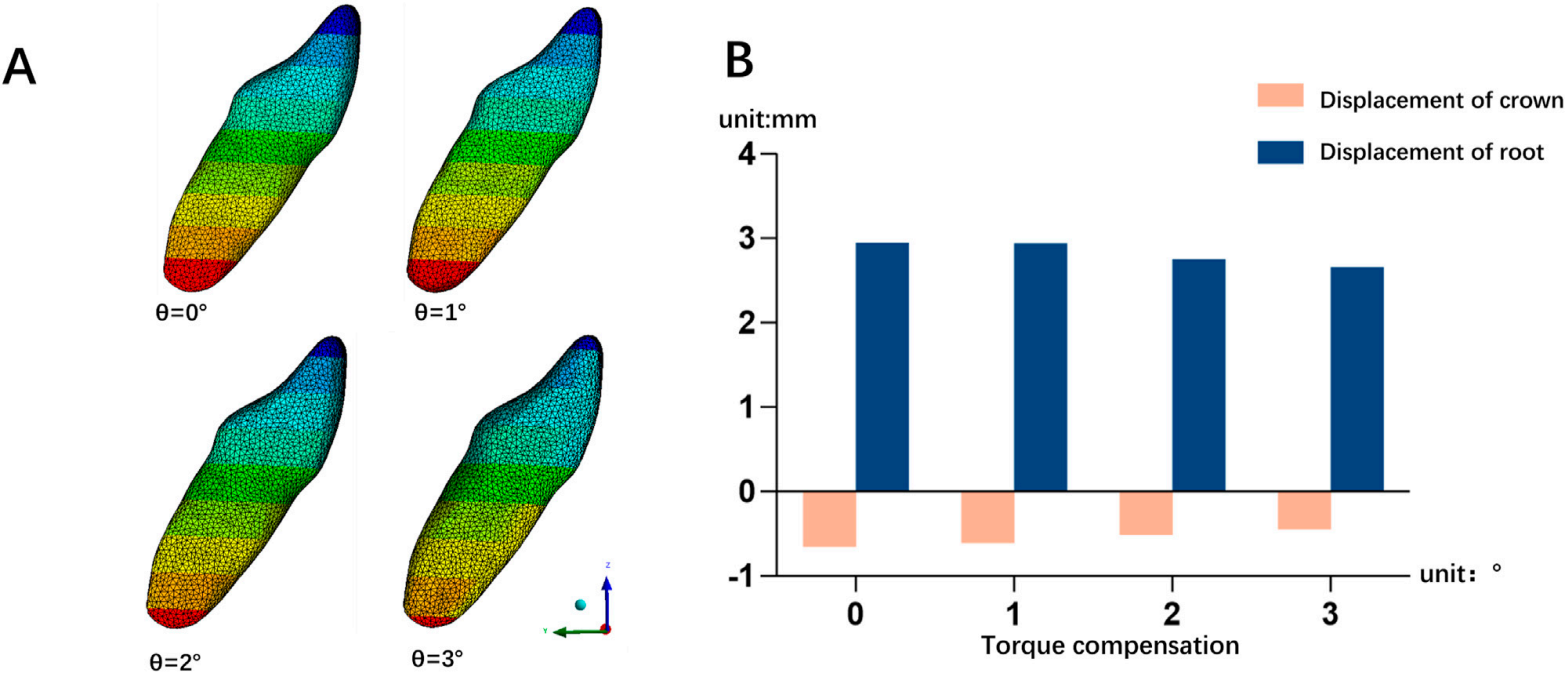
**(A)** The displacement of mandibular right central incisor in different torque compensation groups on the M3-4 model; **(B)** The crown-root movement of mandibular right central incisor in different torque compensation groups on the M3-4 model.

**FIGURE 7:**
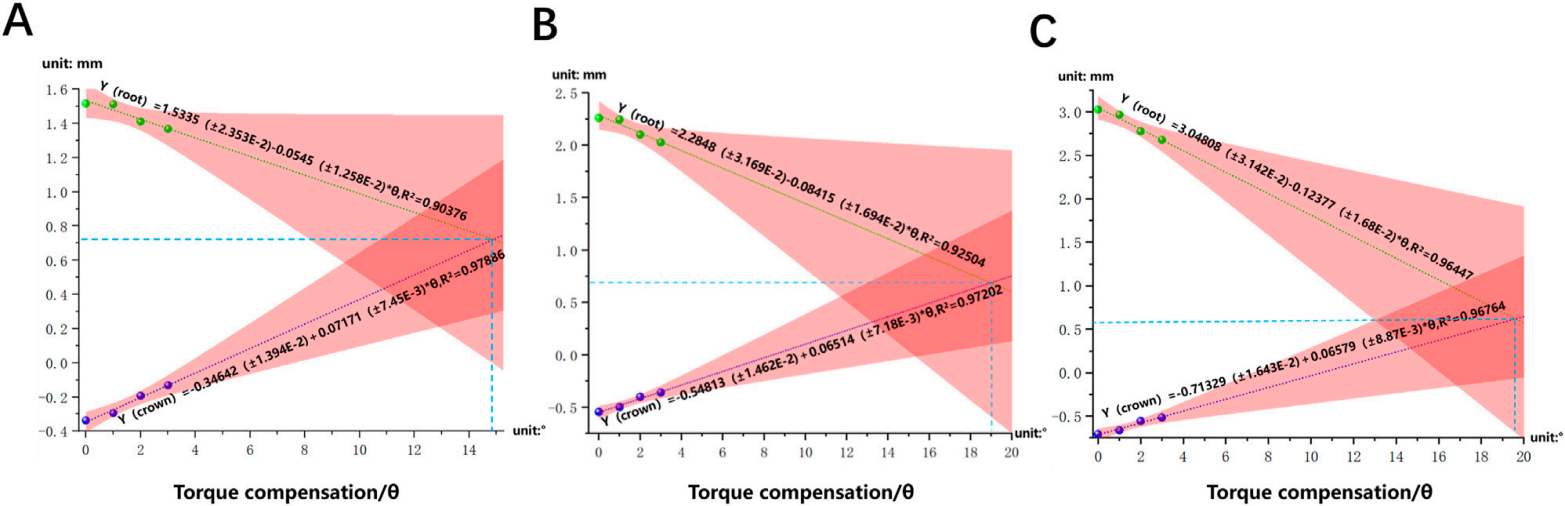
The linear regression model of crown-root displacement of mandibular central incisor and torque per millimeter on the M1-4 **(A)**, M2-3 **(B)** and M3-4 **(C)** model.

**FIGURE 8:**
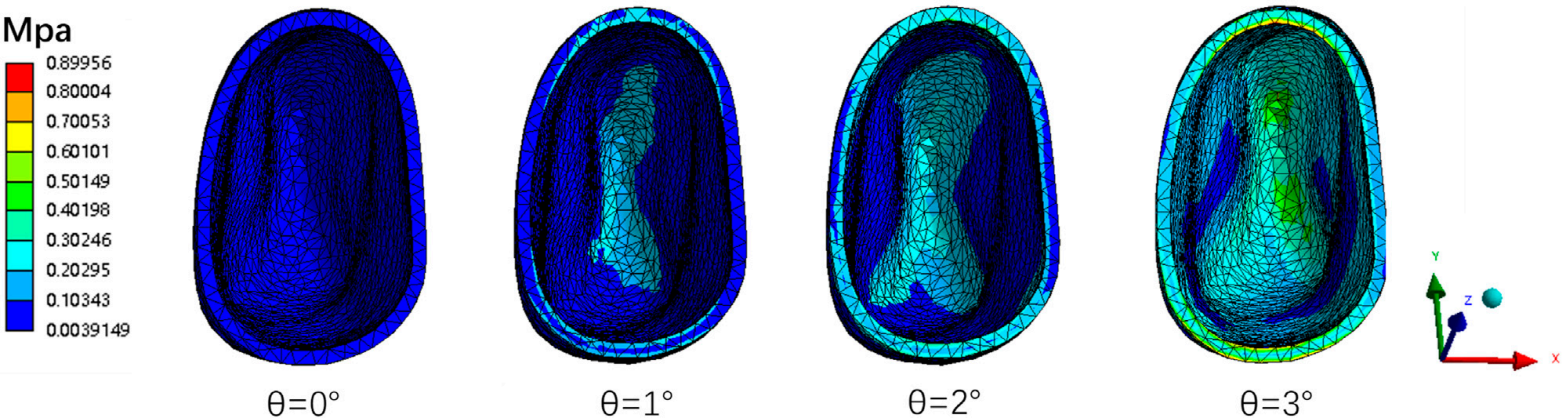
The PDL Von-mises (equivalent) stress of mandibular right central incisor in different torque compensation groups on the M3-4 model.

As the torque compensation increased (0°, 1°, 2°, 3°), the labial inclination of the mandibular central incisor tended to decrease, indicating a transition from translation to torque movement of the lower central incisor (Figures 6A, B). Based on a linear regression model matched to the measured data, it was predicted that when the angle of compensation is approximately 15°, 19°, and 20°, the crown and root of the mandibular central incisor are displaced equally and towards the buccal side, achieving bodily movement, in the three models M1-2, M2-3, and M3-4, respectively (*P*< 0.05) (Figure 7). Furthermore, PDL stress increased significantly with increasing torque compensation, concentrating in the cervical and apical areas (Figure 8).

**FIGURE 9:**
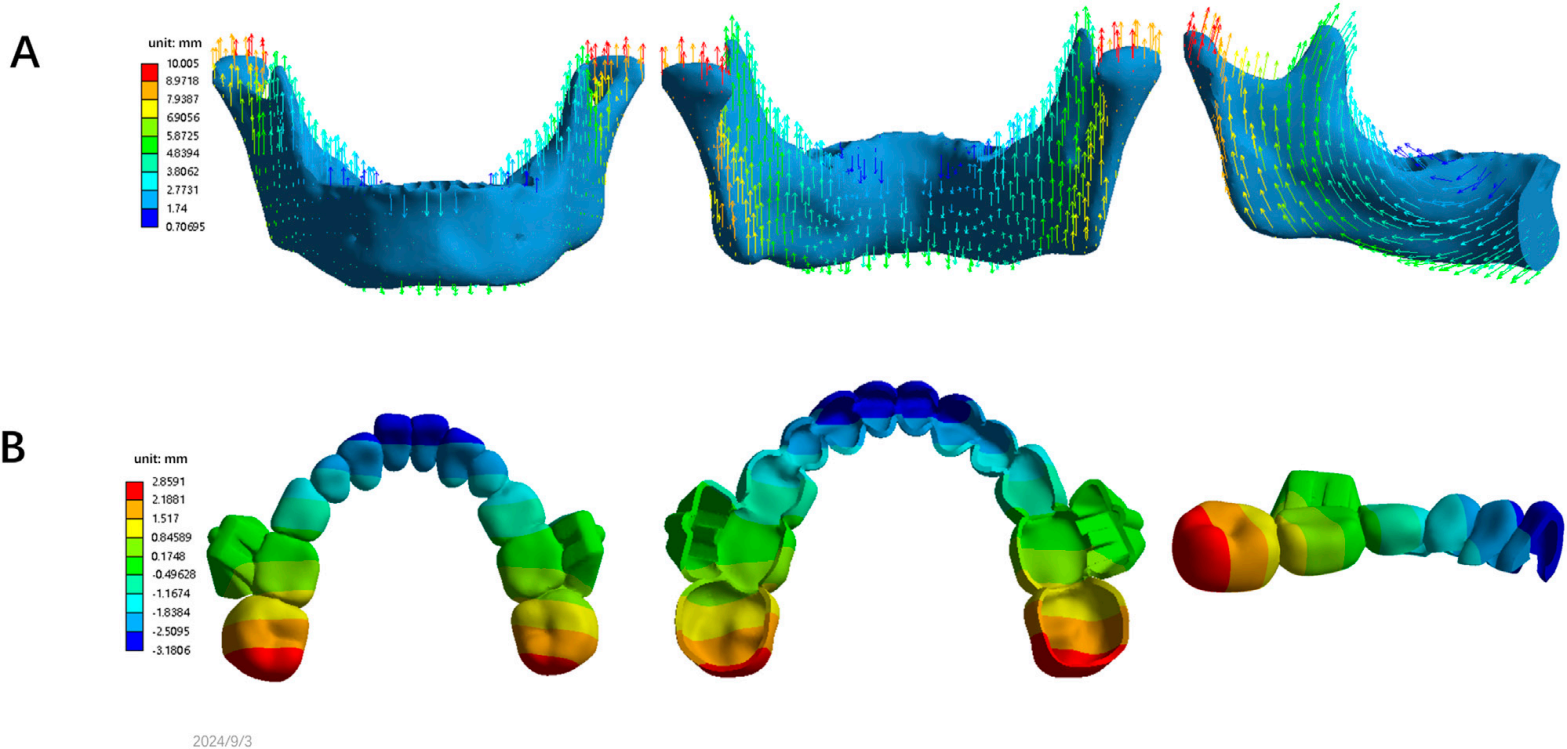
**(A)** The displacement of mandibular bone on the M2-4 model; **(B)** The occlusal displacement of mandibular clear aligner in the M2-4 model.

The personalized CAT for mandibular advancement were associated with a clockwise rotation of the mandibular bone and occlusal plane (Figure 9A). The mandibular clear aligners with personalized buccal wings induced the maximum occlusal displacement in the first molar area during mandibular advancement (Figure 9B).

## 4 Discussion

Advancements in digitization have increased the use of CAT for mandibular advancement in clinical practice. More adolescents are opting for CAT due to its comfort and aesthetic appeal (Lynch et al., 2023). Research indicates that excessive protrusion can adversely affect periodontal membrane reconstruction (Lee et al., 2018), and excessive stress, particularly, at a mandibular advancement of 7 mm, may lead to root absorption (Lee et al., 2018). Therefore, further investigation is needed to determine the optimal amounts of mandibular advancement and occlusal opening.

During mandibular advancement, the temporalis, masseter, and medial pterygoid muscles are activated, generating a posterosuperior force. This force is transmitted to the appliance, teeth, and jawbone via the locking mechanisms (Zhou et al., 2019). Clockwise rotation of the mandible and labial inclination of the mandibular anterior teeth are common during this process, requiring careful consideration in patients with pre-existing labial inclinations in the lower anterior region and those with high-angle cases.

Previous studies have found that clear aligners for mandibular advancement are significantly more effective than traditional orthodontic appliances in controlling lower anterior teeth and the mandibular plane (Wu et al., 2023; Yang et al., 2021; Koukou et al., 2022). However, there are conflicting views regarding the direction of mandibular rotation during mandibular advancement (Yu et al., 2023; Yang et al., 2021), and existing mechanical studies are limited. This study found that as the advancement distance increases, the activity of the DM, MT, and PT muscles intensified, while the activity of the SM and MP muscles weakened.

During mandibular advancement, the altered position of the mandible changes the direction and magnitude of muscle forces, especially those exerted by the masseter, temporalis, and medial pterygoid muscles (Akita et al., 2019). These changes are necessary to adapt to the new mandibular position and to maintain occlusal balance. Due to differences in anatomical roles and mechanics involved in stabilizing the mandible, the forward forces generated by certain muscle groups decrease as the mandible advances, while those generating backward forces become more significant. This redistribution of muscle activity helps achieve balanced muscle forces across the mandible, reducing excessive strain on specific areas. For instance, as the mandible advances, the superficial masseter fibers which are less aligned to generate force in this new forward position, become less active. Conversely, the deep masseter, with its deeper and more vertical fiber orientation, is better suited to stabilize and guide the jaw in this advanced position, making it more active (Se et al., 2022). This shift in muscle activity may explain the concentration of stress moving from the mandibular first primary molar to the first molar.

In this study, stress on the posterior PDL during mandibular advancement was significantly higher than that on the anterior PDL, which aligns with findings from other studies (Zhiguo et al., 2023; Lee et al., 2018). We focused on analyzing stress patterns to identify conditions that minimize stress concentration in specific regions, which is crucial for optimizing clinical outcomes. The distribution of stress in the posterior PDL has significant implications for orthodontic treatment, as uneven stress can lead to undesired tooth movements, such as tilting or rotation, or can damage periodontal structures. For example, excessive stress concentrations in the posterior region could increase the risk of root resorption and damage to the periodontal membrane, potentially leading to irreversible changes in the tooth’s support structure. In contrast, an even stress distribution can promote more controlled tooth movements and enhance treatment stability, especially during mandibular advancement (Consolaro et al., 2023). Our study observed that when the mandibular advancement distance was equal or slightly excessed the occlusal opening distance, the stress on posterior PDL decreased and became more evenly distributed. These findings underscore the importance of balancing mandibular advancement distance and occlusal opening distance to ensure a favorable biomechanical environment for tooth movement and maintain periodontal health.

When the ratio of advancement distance to occlusal opening distance was less than 1, the contributions of the designed oral muscles (DM, MT, PT, SM, and MP) were substantial, working together effectively to maintain balance. As this ratio increased, the contributions of the anterior muscle bundle decreased, potentially explaining the observed reduction in stress on the posterior PDL. Conversely, when the ratio exceeded 1, the force generated by the DM, MT, and PT significantly exceeded those of the SM and MP, becoming the dominant forces. As this ratio continued to increase, the influence of the posterior muscle bundle increased significantly, which likely accounts for the increase and concentration of stress on the posterior PDL during this phase. Therefore, to maintain periodontal health during the advancement process, it is advisable for the mandibular advancement distance to be equal to or slightly greater than the occlusal opening distance. For cases requiring additional mandibular advancement, phased advancement using clear aligners is recommended to better manage stress distribution and ensure a more gradual adaptation of the surrounding structures.

This study also demonstrated that when the sum of advancement distance and occlusal opening distance remained constant, an increase in the occlusal opening distance significantly raised the stress on the posterior PDL and the labial inclination of mandibular anterior teeth. This excessive stress could potentially lead to root absorption (Lee et al., 2018). Moreover, the labial inclination of the lower incisors increases the risk of gingival recession, bone fenestration or dehiscence (Kraus et al., 2014), and it amplifies dental compensatory effects (Wu et al., 2023). Therefore, controlling the occlusal opening distance is crucial for maintaining the periodontal health of both anterior and posterior teeth. CAT offer an advantage in this regard, as they can eliminate occlusal interferences before advancement and require less occlusal opening distance than traditional appliances (Wang and Liu, 2021). The buccal wings of clear aligners, in comparison to bite ramps used in traditional appliances, can also prevent excessive bite opening, which may explain their better control over the labial inclination of the lower anterior teeth in clinical practice. Clear aligners with buccal wings may be suitable for patients with skeletal Class II malocclusion and occlusal interferences.

Recent research has shown that torque compensation can reduce the buccal inclination of posterior teeth during maxillary expansion with clear aligners (Yao et al., 2023). However, some degree of labial inclination of the lower incisors during mandibular advancement is inevitable, which can have significant adverse effects (Bartolucci et al., 2019; Li et al., 2017). It increases the likelihood of periodontal disease, gingival recession, bone dehiscence, and even reduces the skeletal effects of mandibular advancement (Bishara, 2006; Morris et al., 2017; Sulewska et al., 2021). Torque compensation can reduce the labial movement of the lower anterior crowns and the lingual movement of the roots. Therefore, the labio-lingual control of the lower anterior teeth during the mandibular advancement process is very important. In this study, to enhance control over mandibular anterior teeth, torque compensation was applied to the lower central incisor. As the torque compensation increased, the labial inclination of the central mandibular incisor tended to decrease. In models M1-2, M2-3 or M3-4, regression analysis predicted that torque compensation for the lower central incisor at approximately 15°, 19° or 20°, respectively, resulted in nearly bodily movement of the incisor. Nevertheless, studies have suggested that the effectiveness of torque movements with clear aligners may fall short of predictions. Hong et al. found that the actual achieved torque movement was significantly lower than anticipated, with a mean efficiency of 46.81% ± 33.95% (Hong et al., 2023). Similarly, Rajan et al. reported that the accuracy of achieved lower incisor lingual root torque was around 58.2% when ≥10° of torque change was planned (Rajan et al., 2024). These findings suggest that overcompensation may be necessary to achieve the desired torque outcomes. Control of incisor inclination is achieved through the combined action of the crown lingual force generated by torque compensation and the crown labial force exerted by the clear aligners, resembling a couple force.

We concentrated on the analysis of the M3-4 model because all three models displayed the same pattern of increased torque compensation. Our study found that stress on the PDL in the lower central incisor increases with increasing torque compensation, focusing on the cervical and apical regions of the PDL. The movement of the lower central incisor transitioned into torque movement, which particularly suitable for patients with mandibular retrognathia with anterior labial inclination. Thus, appropriate torque control is essential to reduce excessive labial inclination. However, excessive stress may cause external root resorption (Chan et al., 2004). If the torque compensation is too large, it may be detrimental to periodontal health. To prevent root resorption with excessive torque compensation, it is necessary to monitor the relationship between teeth and alveolar bone after loading. Additionally, mandibular anchorage screws, anterior lip-side voids, power-ridge, and lip muscle training should be employed to control the labial inclination of interior teeth during mandibular advancemnt (Yu et al., 2023; Yang et al., 2021).

Mandibular clockwise rotation and an increased mandibular plane angle were observed with clear aligners for mandibular advancement, which was consistent with previous clinical studies, with mandibular plane angle increases by approximately 1.87° (Yang et al., 2021). Therefore, caution should be exercised in mandibular advancement in skeletal Class II high-angle malocclusion. However, some clinical case reports have indicated that the mandibular plane angle is essentially unchanged during mandibular advancement with clear aligners (Yu et al., 2023). This could be due to the occlusal pad of clear aligners contributing to posterior teeth intrusion (Iscan and Sarisoy, 1997; Talens-Cogollos et al., 2022), leading to relative counterclockwise mandibular rotation. In addition, this study found that the maximum occlusal displacement of the mandibular clear aligners occurs on the first molar area. Adding attachments in the first molar area is a recommended strategy to improve aligner fit and prevent unwanted movement.

### 4.1 Limitations and recommendations

Although a significant correlation exists between tooth movement observed in clinical practice and the outcomes of FEA (Wang et al., 2024), it cannot completely replicate the actual oral environment and the properties of materials. Additionally, FEA cannot directly observe the exact biological remodeling of the alveolar bone. In this study, the remodeling direction of periodontal tissues can only be inferred from the stress distribution, which represents a limitation. Enhancing parameters and refining methods are necessary to achieve a more accurate simulation of clinical conditions using FEA. Additionally, while FEA primarily provides static and instantaneous results, further *in vitro* mechanical experiments and *in vivo* clinical trials are necessary to validate and refine these results, especially when determining accurate yield stress. It should be noted that the intrusion effect of the occlusal pad was not considered in this study. To enhance the clinical efficacy of clear aligners for mandibular advancement, more research is needed on the buccal wing’s shape and position.

In this study, we focused on five key muscle groups—superficial masseter, deep masseter, medial and posterior temporalis, and medial pterygoid—due to their significant roles in mandibular stabilization and force generation during mandibular advancement. Although other muscles, such as the lateral pterygoid also contribute to mandibular dynamics, their influence is more related to specific movements. Therefore, the selected muscle groups were considered sufficient for analyzing the primary biomechanical changes during mandibular advancement with clear aligners. Future research may extend the analysis to include these additional muscles for a more comprehensive understanding of muscle interactions during mandibular movement.

## 5 Conclusion and clinical implications

Based on the findings and considering the limitations of this study, we concluded the following.

1. When the mandibular advancement equaled or slightly excessed the occlusal opening distance measurement, the stress on the posterior PDL is reduced and became more evenly distributed. For cases requiring further mandibular advancement, phased advancement using clear aligners is recommended to minimize excessive stress.
2. Increasing occlusal opening distance significantly elevates the stress on the posterior PDL and labial inclination of mandibular anterior teeth. Therefore, careful control of the occlusal opening distance during mandibular advancement is essential for maintain the periodontal health of both anterior and posterior teeth.
3. As torque compensation increases, the labial inclination of the central mandibular incisor tended to decrease, but the stress on the PDL also rises. To prevent root resorption with excessive torque compensation, it is important to monitor of the teeth after loading.
4. Adding attachments in the first molar area is a recommended strategy to enhance aligner fit and prevent unwanted movement, when using clear aligners with buccal wing during mandibular advancement.

## Data Availability

The original contributions presented in the study are included in the article/Supplementary Material, further inquiries can be directed to the corresponding authors.

## Abbreviations

CBCT, Cone-beam computed tomography; CAT, Clear aligner treatment; DM, Deep masseter; FEA, Finite Element Analysis; PDL, Periodontal ligament; SM, Superficial masseter; MT, Middle temporalis and posterior temporalis; MP, Medial pterygoid.
