# Supplementary material for: Biomechanical effects of different mandibular movements and torque compensations during mandibular advancement with clear aligners: a finite element analysis
Source: Front Bioeng Biotechnol. 2024 Nov 13;12:1496517. doi: 10.3389/fbioe.2024.1496517 (PMC11603354; doi:10.3389/fbioe.2024.1496517)
Supplement: Supplementary file 1 [file Table1.DOCX]

Supplementary Material

# Supplementary Figures and Tables

## Supplementary Tables

Taking the right side of the mandibular model as an example, all tables show the forces and directions of SM, DM, MT, PT, and MP of different advancement distance(1-7mm) and occlusal opening distance (2-4mm). SM, superficial masseter; DM, deep masseter; PT, posterior temporalis; MT, middle temporalis; MP, medial pterygoid.

| Table 1. occlusal opening distance (2mm) | | | | | | | |
| --- | --- | --- | --- | --- | --- | --- | --- |
| SM advancement distance | cosx | cosy | cosz | the value of x direction(N) | the value of y direction(N) | the value of z direction(N) | the sum value(N) |
| 1mm | 0.203 | 0.395 | 0.896 | -0.113 | -0.221 | 0.500 | 0.558 |
|  |  |  |  |  |  |  |  |
| 2mm | 0.204 | 0.381 | 0.902 | -0.079 | -0.147 | 0.347 | 0.385 |
|  |  |  |  |  |  |  |  |
| 3mm | 0.205 | 0.368 | 0.907 | -0.043 | -0.078 | 0.192 | 0.211 |
|  |  |  |  |  |  |  |  |
| 4mm | 0.206 | 0.354 | 0.912 | -0.008 | -0.013 | 0.035 | 0.038 |
|  |  |  |  |  |  |  |  |
| 5mm | - | - | - | 0.000 | 0.000 | 0.000 | 0.000 |
|  |  |  |  |  |  |  |  |
| 6mm | - | - | - | 0.000 | 0.000 | 0.000 | 0.000 |
|  |  |  |  |  |  |  |  |
| 7mm | - | - | - | 0.000 | 0.000 | 0.000 | 0.000 |

**Supplementary Figure 1**. The forces and directions of SM in the three-dimensional direction of different advancement distance(1-7mm), with an occlusal opening distance of 2 mm. SM: superficial masseter.

| Table 2. occlusal opening distance (3mm) | | | | | | | | |
| --- | --- | --- | --- | --- | --- | --- | --- | --- |
| SM advancement distance | | cosx | cosy | cosz | the value of x direction(N) | the value of y direction(N) | the value of z direction(N) | the sum value(N) |
| 1mm | 0.200 | | 0.389 | 0.899 | -0.185 | -0.360 | 0.831 | 0.924 |
|  |  | |  |  |  |  |  |  |
| 2mm | 0.201 | | 0.376 | 0.905 | -0.151 | -0.282 | 0.680 | 0.751 |
|  |  | |  |  |  |  |  |  |
| 3mm | 0.202 | | 0.363 | 0.910 | -0.117 | -0.210 | 0.526 | 0.577 |
|  |  | |  |  |  |  |  |  |
| 4mm | 0.203 | | 0.349 | 0.915 | -0.082 | -0.141 | 0.370 | 0.404 |
|  |  | |  |  |  |  |  |  |
| 5mm | 0.204 | | 0.335 | 0.920 | -0.047 | -0.077 | 0.212 | 0.230 |
|  |  | |  |  |  |  |  |  |
| 6mm | 0.205 | | 0.321 | 0.925 | -0.012 | -0.018 | 0.053 | 0.057 |
|  |  | |  |  |  |  |  |  |
| 7mm | - | | - | - | 0.000 | 0.000 | 0.000 | 0.000 |

**Supplementary Figure 2**. The forces and directions of SM in the three-dimensional direction of different advancement distance(1-7mm), with an occlusal opening distance of 3 mm. SM: superficial masseter**.**

| Table 3. occlusal opening distance (4mm) | | | | | | | | |
| --- | --- | --- | --- | --- | --- | --- | --- | --- |
| SM advancement distance | | cosx | cosy | cosz | the value of x direction(N) | the value of y direction(N) | the value of z direction(N) | the sum value(N) |
| 1mm | 0.198 | | 0.385 | 0.906 | -0.256 | -0.497 | 1.169 | 1.291 |
|  |  | |  |  |  |  |  |  |
| 2mm | 0.199 | | 0.372 | 0.911 | -0.222 | -0.416 | 1.018 | 1.117 |
|  |  | |  |  |  |  |  |  |
| 3mm | 0.200 | | 0.359 | 0.916 | -0.189 | -0.339 | 0.864 | 0.943 |
|  |  | |  |  |  |  |  |  |
| 4mm | 0.200 | | 0.344 | 0.917 | -0.154 | -0.265 | 0.706 | 0.770 |
|  |  | |  |  |  |  |  |  |
| 5mm | 0.201 | | 0.332 | 0.922 | -0.120 | -0.198 | 0.550 | 0.596 |
|  |  | |  |  |  |  |  |  |
| 6mm | 0.202 | | 0.317 | 0.927 | -0.085 | -0.134 | 0.392 | 0.423 |
|  |  | |  |  |  |  |  |  |
| 7mm | 0.202 | | 0.302 | 0.930 | -0.050 | -0.075 | 0.232 | 0.249 |

**Supplementary Figure 3**. The forces and directions of SM in the three-dimensional direction of different advancement distance(1-7mm), with an occlusal opening distance of 4 mm. SM: superficial masseter.

| Table 4. occlusal opening distance (2mm) | | | | | | | |
| --- | --- | --- | --- | --- | --- | --- | --- |
| DM advancement distance | cosx | cosy | cosz | the value of x direction(N) | the value of y direction(N) | the value of z direction(N) | the sum value(N) |
| 1mm | 0.510 | 0.369 | 0.777 | -0.793 | 0.574 | 1.208 | 1.555 |
|  |  |  |  |  |  |  |  |
| 2mm | 0.503 | 0.398 | 0.767 | -0.932 | 0.737 | 1.420 | 1.852 |
|  |  |  |  |  |  |  |  |
| 3mm | 0.496 | 0.426 | 0.756 | -1.066 | 0.916 | 1.625 | 2.149 |
|  |  |  |  |  |  |  |  |
| 4mm | 0.489 | 0.453 | 0.745 | -1.196 | 1.108 | 1.822 | 2.446 |
|  |  |  |  |  |  |  |  |
| 5mm | 0.481 | 0.479 | 0.734 | -1.320 | 1.314 | 2.014 | 2.743 |
|  |  |  |  |  |  |  |  |
| 6mm | 0.474 | 0.504 | 0.722 | -1.441 | 1.532 | 2.195 | 3.041 |
|  |  |  |  |  |  |  |  |
| 7mm | 0.466 | 0.527 | 0.711 | -1.555 | 1.759 | 2.373 | 3.338 |

**Supplementary Figure 4**. The forces and directions of DM in the three-dimensional direction of different advancement distance(1-7mm), with an occlusal opening distance of 2 mm. DM: deep masseter

| Table 5. occlusal opening distance (3mm) | | | | | | | |
| --- | --- | --- | --- | --- | --- | --- | --- |
| DM advancement distance | cosx | cosy | cosz | the value of x direction(N) | the value of y direction(N) | the value of z direction(N) | the sum value(N) |
| 1mm | 0.496 | 0.359 | 0.790 | -1.083 | 0.784 | 1.725 | 2.184 |
|  |  |  |  |  |  |  |  |
| 2mm | 0.490 | 0.388 | 0.781 | -1.216 | 0.963 | 1.938 | 2.481 |
|  |  |  |  |  |  |  |  |
| 3mm | 0.484 | 0.416 | 0.770 | -1.345 | 1.156 | 2.139 | 2.778 |
|  |  |  |  |  |  |  |  |
| 4mm | 0.477 | 0.442 | 0.760 | -1.467 | 1.359 | 2.337 | 3.075 |
|  |  |  |  |  |  |  |  |
| 5mm | 0.470 | 0.468 | 0.748 | -1.585 | 1.578 | 2.522 | 3.372 |
|  |  |  |  |  |  |  |  |
| 6mm | 0.463 | 0.492 | 0.737 | -1.699 | 1.805 | 2.704 | 3.669 |
|  |  |  |  |  |  |  |  |
| 7mm | 0.456 | 0.515 | 0.726 | -1.809 | 2.043 | 2.880 | 3.967 |

**Supplementary Figure 5**. The forces and directions of DM in the three-dimensional direction of different advancement distance(1-7mm), with an occlusal opening distance of 3 mm. DM: deep masseter

| Table 6. occlusal opening distance (4mm) | | | | | | | |
| --- | --- | --- | --- | --- | --- | --- | --- |
| DM advancement distance | cosx | cosy | cosz | the value of x direction(N) | the value of y direction(N) | the value of z direction(N) | the sum value(N) |
| 1mm | 0.483 | 0.349 | 0.802 | -1.359 | 0.982 | 2.256 | 2.813 |
|  |  |  |  |  |  |  |  |
| 2mm | 0.478 | 0.378 | 0.793 | -1.487 | 1.176 | 2.466 | 3.110 |
|  |  |  |  |  |  |  |  |
| 3mm | 0.472 | 0.405 | 0.783 | -1.608 | 1.380 | 2.668 | 3.407 |
|  |  |  |  |  |  |  |  |
| 4mm | 0.465 | 0.432 | 0.773 | -1.722 | 1.600 | 2.863 | 3.704 |
|  |  |  |  |  |  |  |  |
| 5mm | 0.459 | 0.457 | 0.762 | -1.837 | 1.829 | 3.049 | 4.001 |
|  |  |  |  |  |  |  |  |
| 6mm | 0.452 | 0.481 | 0.751 | -1.939 | 2.064 | 3.222 | 4.290 |
|  |  |  |  |  |  |  |  |
| 7mm | 0.446 | 0.504 | 0.740 | -2.050 | 2.316 | 3.401 | 4.595 |

**Supplementary Figure 6**. The forces and directions of DM in the three-dimensional direction of different advancement distance(1-7mm), with an occlusal opening distance of 4 mm. DM: deep masseter.

| Table 7. occlusal opening distance (2mm) | | | | | | | |
| --- | --- | --- | --- | --- | --- | --- | --- |
| MT advancement distance | cosx | cosy | cosz | the value of x direction(N) | the value of y direction(N) | the value of z direction(N) | the sum value(N) |
| 1mm | 0.216 | 0.499 | 0.839 | -0.209 | 0.482 | 0.810 | 0.965 |
|  |  |  |  |  |  |  |  |
| 2mm | 0.215 | 0.508 | 0.834 | -0.255 | 0.603 | 0.990 | 1.187 |
|  |  |  |  |  |  |  |  |
| 3mm | 0.214 | 0.516 | 0.829 | -0.302 | 0.727 | 1.168 | 1.409 |
|  |  |  |  |  |  |  |  |
| 4mm | 0.213 | 0.524 | 0.824 | -0.347 | 0.855 | 1.344 | 1.631 |
|  |  |  |  |  |  |  |  |
| 5mm | 0.211 | 0.532 | 0.820 | -0.392 | 0.986 | 1.520 | 1.853 |
|  |  |  |  |  |  |  |  |
| 6mm | 0.210 | 0.540 | 0.815 | -0.436 | 1.121 | 1.691 | 2.075 |
|  |  |  |  |  |  |  |  |
| 7mm | 0.209 | 0.548 | 0.810 | -0.480 | 1.259 | 1.861 | 2.297 |

**Supplementary Figure 7**. The forces and directions of MT in the three-dimensional direction of different advancement distance(1-7mm), with an occlusal opening distance of 2 mm. MT: middle temporalis.

| Table 8. occlusal opening distance (3mm) | | | | | | | |
| --- | --- | --- | --- | --- | --- | --- | --- |
| MT advancement distance | cosx | cosy | cosz | the value of x direction(N) | the value of y direction(N) | the value of z direction(N) | the sum value(N) |
| 1mm | 0.214 | 0.494 | 0.842 | -0.286 | 0.660 | 1.126 | 1.337 |
|  |  |  |  |  |  |  |  |
| 2mm | 0.213 | 0.503 | 0.838 | -0.332 | 0.784 | 1.306 | 1.559 |
|  |  |  |  |  |  |  |  |
| 3mm | 0.212 | 0.511 | 0.833 | -0.378 | 0.910 | 1.484 | 1.781 |
|  |  |  |  |  |  |  |  |
| 4mm | 0.211 | 0.519 | 0.828 | -0.423 | 1.040 | 1.658 | 2.003 |
|  |  |  |  |  |  |  |  |
| 5mm | 0.210 | 0.527 | 0.823 | -0.467 | 1.173 | 1.831 | 2.225 |
|  |  |  |  |  |  |  |  |
| 6mm | 0.208 | 0.535 | 0.819 | -0.509 | 1.309 | 2.004 | 2.447 |
|  |  |  |  |  |  |  |  |
| 7mm | 0.207 | 0.543 | 0.814 | -0.552 | 1.449 | 2.173 | 2.669 |

**Supplementary Figure 8**. The forces and directions of MT in the three-dimensional direction of different advancement distance(1-7mm), with an occlusal opening distance of 3 mm. MT: middle temporalis.

| Table 9. occlusal opening distance (4mm) | | | | | | | |
| --- | --- | --- | --- | --- | --- | --- | --- |
| MT advancement distance | cosx | cosy | cosz | the value of x direction(N) | the value of y direction(N) | the value of z direction(N) | the sum value(N) |
| 1mm | 0.212 | 0.490 | 0.846 | -0.362 | 0.837 | 1.446 | 1.709 |
|  |  |  |  |  |  |  |  |
| 2mm | 0.211 | 0.498 | 0.841 | -0.407 | 0.961 | 1.624 | 1.931 |
|  |  |  |  |  |  |  |  |
| 3mm | 0.210 | 0.506 | 0.836 | -0.452 | 1.089 | 1.800 | 2.153 |
|  |  |  |  |  |  |  |  |
| 4mm | 0.209 | 0.515 | 0.832 | -0.496 | 1.223 | 1.976 | 2.375 |
|  |  |  |  |  |  |  |  |
| 5mm | 0.208 | 0.523 | 0.826 | -0.540 | 1.358 | 2.145 | 2.597 |
|  |  |  |  |  |  |  |  |
| 6mm | 0.206 | 0.531 | 0.822 | -0.581 | 1.497 | 2.317 | 2.819 |
|  |  |  |  |  |  |  |  |
| 7mm | 0.205 | 0.538 | 0.817 | -0.623 | 1.636 | 2.484 | 3.041 |

**Supplementary Figure 9**. The forces and directions of MT in the three-dimensional direction of different advancement distance(1-7mm), with an occlusal opening distance of 4 mm. MT: middle temporalis.

| Table 10. occlusal opening distance (2mm) | | | | | | | |
| --- | --- | --- | --- | --- | --- | --- | --- |
| PT advancement distance | cosx | cosy | cosz | the value of x direction(N) | the value of y direction(N) | the value of z direction(N) | the sum value (N) |
| 1mm | 0.204 | 0.850 | 0.485 | -0.212 | 0.883 | 0.504 | 1.039 |
|  |  |  |  |  |  |  |  |
| 2mm | 0.203 | 0.853 | 0.481 | -0.311 | 1.307 | 0.737 | 1.532 |
|  |  |  |  |  |  |  |  |
| 3mm | 0.201 | 0.855 | 0.477 | -0.407 | 1.731 | 0.966 | 2.025 |
|  |  |  |  |  |  |  |  |
| 4mm | 0.199 | 0.858 | 0.474 | -0.501 | 2.160 | 1.193 | 2.517 |
|  |  |  |  |  |  |  |  |
| 5mm | 0.198 | 0.860 | 0.470 | -0.596 | 2.589 | 1.415 | 3.010 |
|  |  |  |  |  |  |  |  |
| 6mm | 0.196 | 0.863 | 0.466 | -0.687 | 3.023 | 1.632 | 3.503 |
|  |  |  |  |  |  |  |  |
| 7mm | 0.195 | 0.865 | 0.462 | -0.779 | 3.456 | 1.846 | 3.996 |

**Supplementary Figure 10**. The forces and directions of PT in the three-dimensional direction of different advancement distance(1-7mm), with an occlusal opening distance of 2 mm. PT: posterior temporalis.

| Table 11. occlusal opening distance (3mm) | | | | | | | |
| --- | --- | --- | --- | --- | --- | --- | --- |
| PT advancement distance | cosx | cosy | cosz | the value of x direction(N) | the value of y direction(N) | the value of z direction(N) | the sum value(N) |
| 1mm | 0.203 | 0.846 | 0.493 | -0.266 | 1.110 | 0.647 | 1.312 |
|  |  |  |  |  |  |  |  |
| 2mm | 0.202 | 0.849 | 0.489 | -0.365 | 1.532 | 0.883 | 1.805 |
|  |  |  |  |  |  |  |  |
| 3mm | 0.200 | 0.851 | 0.485 | -0.460 | 1.955 | 1.114 | 2.298 |
|  |  |  |  |  |  |  |  |
| 4mm | 0.198 | 0.854 | 0.481 | -0.553 | 2.383 | 1.342 | 2.791 |
|  |  |  |  |  |  |  |  |
| 5mm | 0.197 | 0.857 | 0.477 | -0.647 | 2.814 | 1.566 | 3.283 |
|  |  |  |  |  |  |  |  |
| 6mm | 0.195 | 0.859 | 0.473 | -0.736 | 3.244 | 1.786 | 3.776 |
|  |  |  |  |  |  |  |  |
| 7mm | 0.194 | 0.861 | 0.469 | -0.828 | 3.675 | 2.002 | 4.269 |

**Supplementary Figure 11**. The forces and directions of PT in the three-dimensional direction of different advancement distance(1-7mm), with an occlusal opening distance of 3 mm. PT: posterior temporalis.

| Table 12. occlusal opening distance (4mm) | | | | | | | |
| --- | --- | --- | --- | --- | --- | --- | --- |
| PT advancement distance | cosx | cosy | cosz | the value of x direction(N) | the value of y direction(N) | the value of z direction(N) | the sum value(N) |
| 1mm | 0.202 | 0.842 | 0.500 | -0.320 | 1.335 | 0.793 | 1.586 |
|  |  |  |  |  |  |  |  |
| 2mm | 0.201 | 0.845 | 0.496 | -0.418 | 1.756 | 1.031 | 2.078 |
|  |  |  |  |  |  |  |  |
| 3mm | 0.199 | 0.847 | 0.492 | -0.512 | 2.178 | 1.265 | 2.571 |
|  |  |  |  |  |  |  |  |
| 4mm | 0.198 | 0.850 | 0.488 | -0.607 | 2.604 | 1.495 | 3.064 |
|  |  |  |  |  |  |  |  |
| 5mm | 0.196 | 0.853 | 0.484 | -0.697 | 3.034 | 1.721 | 3.556 |
|  |  |  |  |  |  |  |  |
| 6mm | 0.194 | 0.855 | 0.480 | -0.786 | 3.462 | 1.944 | 4.049 |
|  |  |  |  |  |  |  |  |
| 7mm | 0.193 | 0.858 | 0.477 | -0.877 | 3.897 | 2.166 | 4.542 |

**Supplementary Figure 12**. The forces and directions of PT in the three-dimensional direction of different advancement distance(1-7mm), with an occlusal opening distance of 4 mm. PT: posterior temporalis.

| Table 13. occlusal opening distance (2mm) | | | | | | | |
| --- | --- | --- | --- | --- | --- | --- | --- |
| MP advancement distance | cosx | cosy | cosz | the value of x direction(N) | the value of y direction(N) | the value of z direction(N) | the sum value(N) |
| 1mm | 0.473 | 0.342 | 0.812 | 0.271 | -0.196 | 0.465 | 0.573 |
|  |  |  |  |  |  |  |  |
| 2mm | 0.476 | 0.323 | 0.818 | 0.189 | -0.128 | 0.324 | 0.396 |
|  |  |  |  |  |  |  |  |
| 3mm | 0.480 | 0.304 | 0.823 | 0.105 | -0.067 | 0.181 | 0.219 |
|  |  |  |  |  |  |  |  |
| 4mm | 0.483 | 0.284 | 0.829 | 0.021 | -0.012 | 0.035 | 0.043 |
|  |  |  |  |  |  |  |  |
| 5mm | - | - | - | 0.000 | 0.000 | 0.000 | 0.000 |
|  |  |  |  |  |  |  |  |
| 6mm | - | - | - | 0.000 | 0.000 | 0.000 | 0.000 |
|  |  |  |  |  |  |  |  |
| 7mm | - | - | - | 0.000 | 0.000 | 0.000 | 0.000 |

**Supplementary Figure 13**. The forces and directions of MP in the three-dimensional direction of different advancement distance(1-7mm), with an occlusal opening distance of 2 mm. MP: medial pterygoid.

| Table 14. occlusal opening distance (3mm) | | | | | | | |
| --- | --- | --- | --- | --- | --- | --- | --- |
| MP advancement distance | cosx | cosy | cosz | the value of x direction(N) | the value of y direction(N) | the value of z direction(N) | the sum value(N) |
| 1mm | 0.465 | 0.336 | 0.819 | 0.441 | -0.318 | 0.776 | 0.948 |
|  |  |  |  |  |  |  |  |
| 2mm | 0.468 | 0.317 | 0.825 | 0.361 | -0.244 | 0.636 | 0.771 |
|  |  |  |  |  |  |  |  |
| 3mm | 0.471 | 0.298 | 0.830 | 0.280 | -0.177 | 0.493 | 0.594 |
|  |  |  |  |  |  |  |  |
| 4mm | 0.474 | 0.279 | 0.835 | 0.198 | -0.116 | 0.349 | 0.417 |
|  |  |  |  |  |  |  |  |
| 5mm | 0.477 | 0.259 | 0.840 | 0.115 | -0.062 | 0.202 | 0.241 |
|  |  |  |  |  |  |  |  |
| 6mm | 0.479 | 0.239 | 0.844 | 0.031 | -0.015 | 0.054 | 0.064 |
|  |  |  |  |  |  |  |  |
| 7mm | - | - | - | 0.000 | 0.000 | 0.000 | 0.000 |

**Supplementary Figure 14**. The forces and directions of MP in the three-dimensional direction of different advancement distance(1-7mm), with an occlusal opening distance of 3 mm. MP: medial pterygoid.

| Table 15. occlusal opening distance (4mm) | | | | | | | |
| --- | --- | --- | --- | --- | --- | --- | --- |
| MP advancement distance | cosx | cosy | cosz | the value of x direction(N) | the value of y direction(N) | the value of z direction(N) | the sum value(N) |
| 1mm | 0.457 | 0.330 | 0.826 | 0.604 | -0.436 | 1.092 | 1.322 |
|  |  |  |  |  |  |  |  |
| 2mm | 0.460 | 0.312 | 0.831 | 0.527 | -0.357 | 0.952 | 1.146 |
|  |  |  |  |  |  |  |  |
| 3mm | 0.463 | 0.293 | 0.836 | 0.449 | -0.284 | 0.810 | 0.969 |
|  |  |  |  |  |  |  |  |
| 4mm | 0.466 | 0.274 | 0.841 | 0.369 | -0.217 | 0.666 | 0.792 |
|  |  |  |  |  |  |  |  |
| 5mm | 0.468 | 0.255 | 0.846 | 0.288 | -0.157 | 0.521 | 0.615 |
|  |  |  |  |  |  |  |  |
| 6mm | 0.471 | 0.235 | 0.850 | 0.207 | -0.103 | 0.373 | 0.439 |
|  |  |  |  |  |  |  |  |
| 7mm | 0.473 | 0.215 | 0.854 | 0.124 | -0.056 | 0.224 | 0.262 |

**Supplementary Figure 15**. The forces and directions of MP in the three-dimensional direction of different advancement distance(1-7mm), with an occlusal opening distance of 4 mm. MP: medial pterygoid.
